# Metabolic profiling of fatty acids in *Tripterygium wilfordii* multiglucoside- and triptolide-induced liver-injured rats

**DOI:** 10.1515/biol-2021-0016

**Published:** 2021-02-20

**Authors:** Xiaojie Liu, Cong Hu, Hongwei Li, Linjing Wu, Yinhua Xiong, Xilan Tang, Siyu Deng

**Affiliations:** Jiangxi Provincial Key Laboratory of Drug Design and Evaluation, School of Pharmacy, Jiangxi Science and Technology Normal University, Nanchang 330013, P. R. China

**Keywords:** fatty acids, *Tripterygium wilfordii* multiglucoside, triptolide, metabolic profile, liver injury

## Abstract

*Tripterygium wilfordii* multiglucoside (TWM) is a fat-soluble extract from a Chinese herb *T. wilfordii*, that’s used in treating rheumatoid arthritis, nephrotic syndrome and other skin diseases. Triptolide (TP) is a major active component in TWM. However, clinical applications of TWM are limited by its various toxicities especially hepatotoxicity. In recent studies, it has been reported that drug-induced liver injury (DILI) could induce the disorder of lipid metabolism in animals. Hence, this study focuses on the metabolic profile of fatty acids in TWM- and TP-induced liver-injured rats. In serum and liver tissue, 16 free and 16 esterified fatty acids were measured by gas chromatography coupled with mass spectrometry. Metabolic profile of serum fatty acids in rats with liver injury was identified by multivariate statistical analysis. The fatty acid levels in the serum of TWM- and TP-treated rats significantly decreased, whereas those in the liver tissue of TWM- and TP-treated rats obviously increased when compared with the vehicle-treated rats. Four free fatty acids were identified as candidate biomarkers of TWM- and TP-induced liver injury. Therefore, the targeted metabolomic method may be used as a complementary approach for DILI diagnosis in clinic.

## 1 Introduction

The liver is a primary organ of synthesis and metabolism of fatty acids in vertebrates. It is not surprising that any injury to the liver makes a disruption for the balance of the levels of fatty acids including free fatty acids and esterified fatty acids in the body. It has been reported that the levels of fatty acids could be closely related to various liver diseases such as liver cancer [1], hepatitis C [2] and drug-induced liver injury (DILI) [3]. High level of free fatty acids in the body can induce large amounts of lipids to deposit in hepatic cell, resulting in the damage of cell membrane, mitochondria and lysosomes [4]. The decreased ratio of ω-6 and ω-3 polyunsaturated fatty acids in the body could significantly alleviate chemically induced liver injury [5]. The alterations in the fatty acid concentrations and the metabolic profiling may be examined in animals with hepatic damage, showing that these changes could be used to evaluate biochemical processes and pathological status *in vivo*.

*Tripterygium wilfordii* multiglucoside (TWM) is a fat-soluble mixture including alkaloids, diterpene lactones and triterpenoids extracted from a Chinese herb *T. wilfordii* [6]. The TWM tablet is extensively used for the treatment of rheumatoid arthritis, nephrotic syndrome, systemic lupus erythematosus and other diseases in clinics [7]. However, the further development and clinical application of TWM have been limited due to its various toxicities especially hepatotoxicity [8]. Triptolide (TP), a major active ingredient of diterpene lactone in TWM, showed multiple pharmacological effects such as anti-inflammatory [9], antitumor [10,11] and antifertility [12]. The therapeutic effect of TP is 100–200 times higher than that of TWM, while the toxicity of TWM is stronger than that of TP at the equivalent dose [13]. However, the hepatotoxic mechanism of TWM and TP has not been fully clarified. The hepatotoxic mechanism of TP may include oxidation stress [14,15], lipid peroxidation [16], inhibition of mitochondrial respiratory chain [17], DNA damage and hepatocyte apoptosis [18]. A toxic dose of TP could induce obvious changes in the serum levels of different types of lipids such as total free fatty acids, triglycerides and phospholipids in rats [19]. However, it is still unclear whether TP induces the metabolic profile dynamics of fatty acids in animals with liver injury.

Sex difference in lipid metabolism was also obviously observed in the TP-treated rat liver. The levels of total free fatty acids, triglycerides and total bile acids were much higher in the female rats in comparison to the male rats treated by TP [19]. Therefore, in this study, we introduced a metabolomic approach to observe the changes in the fatty acid metabolic profile of TWM- and TP-treated female rats with liver injury. Free and esterified fatty acids in the serum and liver of rats were quantitatively determined by gas chromatograph-mass spectrometry (GC-MS). Moreover, chemometrics such as principal component analysis (PCA) and partial least squares-discriminant analysis (PLS-DA) were used to discover the discrepancies in the metabolic profiling of fatty acids between control and TWM-/TP-induced liver injured rats. We aimed (1) to identify potential fatty acid biomarkers for TWM- and TP-caused hepatic damage and (2) to explore the correlations between TWM-/TP-induced liver injury and serum fatty acid metabolic profile. These results may provide not only a new method for the study of hepatotoxicity of Chinese herb but also a complementary approach for DILI diagnosis in clinic.

## 2 Materials and methods

### 2.1 Chemical reagents

TWM was obtained from Qianjin Xieli Pharmaceutical Co., Ltd (Hunan, China). TP (>98% pure) was provided by Guilin Sanjin Biologics Co., Ltd (Guanxi, China). Docosahexaenoic acid (C22:6n3), docosanoic acid (C22:0), eicosapentaenoic acid (C20:5n3), eicosatrienoic acid (C20:3n6), arachidonic acid (C20:4n6), arachidic acid (C20:0), nonadecadienoic acid (C19:2n6), γ-linolenic acid (C18:3n6), α-linolenic acid (C18:3n3), linoleic acid (C18:2n6), oleic acid (C18:1n9), *cis*-vaccenic acid (C18:1n7), stearic acid (C18:0), palmitic acid (C16:0), palmitoleic acid (C16:1n7), myristic acid (C14:0) and lauric acid (C12:0) were purchased from Nu-Chek Prep (Elysian, MN, USA). Their purities were above 98%. Trimethylsilane diazomethane (TMSCHN_2_) in *n*-hexane (2 mol/L) was purchased from Energy Chemical (Shanghai, China). The other chemical reagents were purchased from Xilong Scientific Co., Ltd (Guangdong, China).

### 2.2 Chemical analysis of TWM by electrospray ionization quadrupole time-of-flight mass spectrometry (ESI-Q-TOF-MS/MS)

The components of TWM powder were extracted using methanol by the ultrasonic method. The ultra-high performance liquid chromatography (UHPLC) analysis was performed on a Shimadzu system (Kyoto, Japan) that consists of an LC-30AD solvent delivery system, a DGU-20A3 degasser, an SIL-30ACXR autosampler, a CTO-30AC column oven and a CBM-20A controller. TWM ingredients were separated on an Ultimate UHPLC XB C18 column (1.8 µm, 100 mm × 2.1 mm i.d.; Welch Technologies, Shanghai, China) at a flow rate of 0.3 mL/min. The mobile phase comprised 0.1% formic acid in water (solvent A) and acetonitrile (solvent B). The linear gradient elution was utilized with the following step: the initial composition of 85% A and 15% B was changed to 70% A and 30% B in 5 min and then maintained for 15 min, and then, B was increased to 40% in 10 min followed by an increase to 70% in another 10 min. The ESI-Q-TOF-MS/MS detection was carried out on a Triple TOF™ 5600 + system in a positive ion mode (AB SCIX, CA, USA). Ion spray voltage and collision energy were optimized to 4.5 kV and 35 eV, respectively. The duo spray source temperature was set to 550°C. MS/MS data were analyzed using Peak View Software™ 1.2 (AB SCIEX, CA, USA).

### 2.3 Animals and chemical treatment

**Figure A1:**
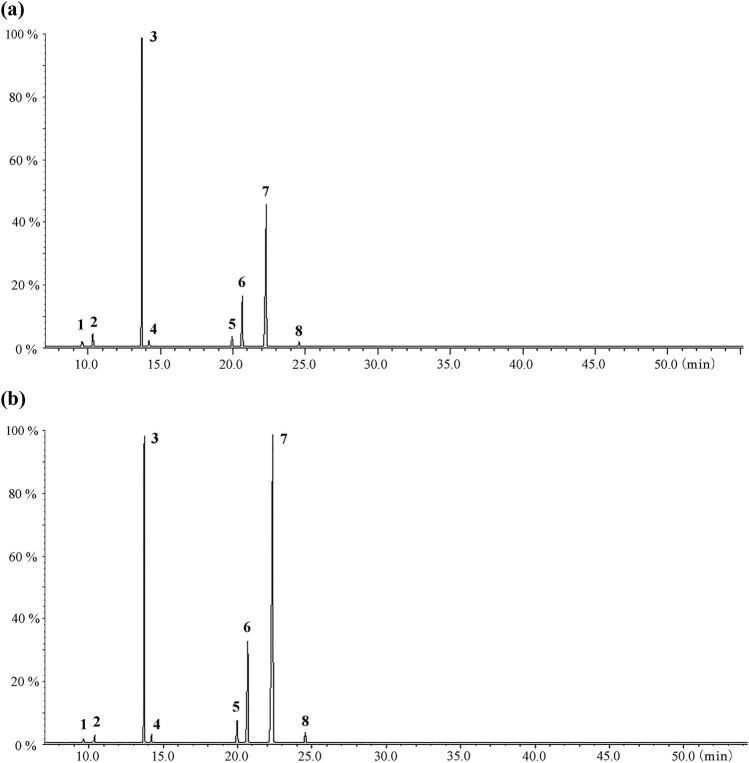
Total ion chromatogram of free fatty acids (a) and esterified fatty acids (b) by GC-MS in the standard rat chow. (1) C12:0, (2) C14:0, (3) C16:0, (4) C16:1n7, (5) C18:0, (6) C18:1n9, (7) C18:1n7 and (8) C18:3n6.

Seven- to nine-week-old female Sprague-Dawley rats weighting 200 ± 20 g (SPF grade, Certificate number: SCXK2016-0002) were purchased from Silaike Jingda Experimental Animal Co., Ltd (Hunan, China). Animals were permitted to acclimatize for 7 days in a 12 h light/dark cycle with regulated temperature and relative humidity. All animals were given free access to standard rat chow (the fatty acid composition is shown in Figure A1) purchased from COFCO Feed Co., Ltd (Hubei, China) and tap water. The animals were randomly assigned to three groups. Control rats (*n* = 10) were administered with distilled water containing 0.5% sodium carboxymethyl cellulose (CMC-Na). TWM-treated rats (*n* = 10) received 3.5 g/kg TWM dissolved in 0.5% CMC-Na. TP-treated rats (*n* = 10) received 2.1 mg/kg TP dissolved in 0.5% CMC-Na. Blood samples were collected 24 h after TWM and TP administration. At the end of the experiments, the rats were sacrificed by euthanasia using pentobarbital sodium, and livers were subsequently collected. All animals were handled in accordance with the standards for laboratory animals (GB14925-2001). The Care and Use of Laboratory Animals protocols were strictly performed.


**Ethical approval:** The research related to animal use has been complied with all the relevant national regulations and institutional policies for the care and use of animals and has been approved by Animal Ethics Committee of Jiangxi Science & Technology Normal University.

### 2.4 Biochemical assays and histopathology

After rat sacrifice, the collected blood was centrifuged at 3,000*g* for 5 min at 4°C to obtain serum. The levels of serum chemical indicators including alanine aminotransferase (ALT), aspartate aminotransferase (AST), alkaline phosphatase (ALP) and total bilirubin (TBIL) were measured using analyte-specific kits (Rongsheng Biotech, Shanghai, China) according to manufacturer’s protocols. Liver sections were randomly selected for histological examination. Slide sections were fixed in 4% paraformaldehyde in 0.1 M phosphate-buffered saline (PBS) for 24 h and routinely embedded in paraffin wax and sliced at 5 µm thickness. After deparaffinization, the sections were stained with hematoxylin and eosin for examination by the microscopy system.

### 2.5 Serum sample preparation for GC-MS analysis

Methyl esterification method of free fatty acids was carried out as follows: 20 µL of internal standard solution (C19:2n6, 1,000 µg/mL) was added to 100 µL of serum. Subsequently, serum was deproteinated with 900 µL solution of chloroform–methanol (2:1, v/v). An aliquot of supernatant (500 µL) was evaporated to dryness under nitrogen. The residue was dissolved in 1,000 µL solution of methanol–toluene (1:1, v/v), and it was methylated using 100 µL trimethylsilyldiazomethane (TMSCHN_2_) in *n*-hexane (2 mol/L) under the condition of vortex for 2 min at 25°C. Finally, the reaction was quenched with 100 µL of acetic acid. The reaction solution was evaporated to dryness under nitrogen and then reconstituted in 500 µL of *n*-hexane. Esterified fatty acids were methylated in the solution of 0.4 M KOH–CH_3_OH according to the method suggested in the previous study [3]. An aliquot (1 µL) of the *n*-hexane solution was injected into the GC-MS system for analysis.

### 2.6 Liver sample preparation for GC-MS analysis

One hundred milligrams of liver sample were homogenized twice by homogenizer (Kinematica, Luzern, Swiss) at 3,000*g* for 30 s with 1,000 µL solution of methanol–chloroform–water (2:1:0.8, v/v/v) under an ice bath condition. The homogenate was centrifuged at 20,000*g* for 15 min at 4°C. After the supernatant being dried under nitrogen, free and esterified fatty acids in the residue were methylated with the same method as serum sample preparation for the GC-MS analysis.

### 2.7 GC-MS analysis

The fatty acid analysis was carried out in a 6890 GC-5973 MS system (Agilent, CA, USA). The analysis conditions can be referred to from the method in the previous study [3]. Briefly, methyl ester of fatty acids was separated on a DB-225MS capillary column (0.25 µm, 60 m × 0.25 mm i.d.; Agilent, CA, USA). The initial temperature of the oven was set at 70°C (held for 1 min), then increased to 200°C at the rate of 40°C/min (held for 20 min) and finally increased to 230°C at the rate of 5°C/min (held for 5 min). The electron impact was operated at 70 eV. Four fragments of *m*/*z* 55, 74, 67 and 79 were simultaneously acquired using selective ion monitoring.

### 2.8 Data processing and statistical analysis

GC-MS data were processed by enhanced MSD ChemStation software (Agilent, CA, USA). PCA and PLS-DA were conducted by SIMCA-P 11.5 edition (Umetrics, Umea, Sweden). Pearson’s correlation analysis was performed using SPSS 18.0 software (SPSS, Chicago, IL, USA). The results for continuous variables were expressed as mean ± standard deviation. Differences between two groups were significantly tested using two-tailed Student’s *t*-tests in SPSS 18.0 software. A *p*-value of <0.05 was considered statistically significant.

## 3 Results

### 3.1 Chemical analysis of TWM

**Figure 1:**
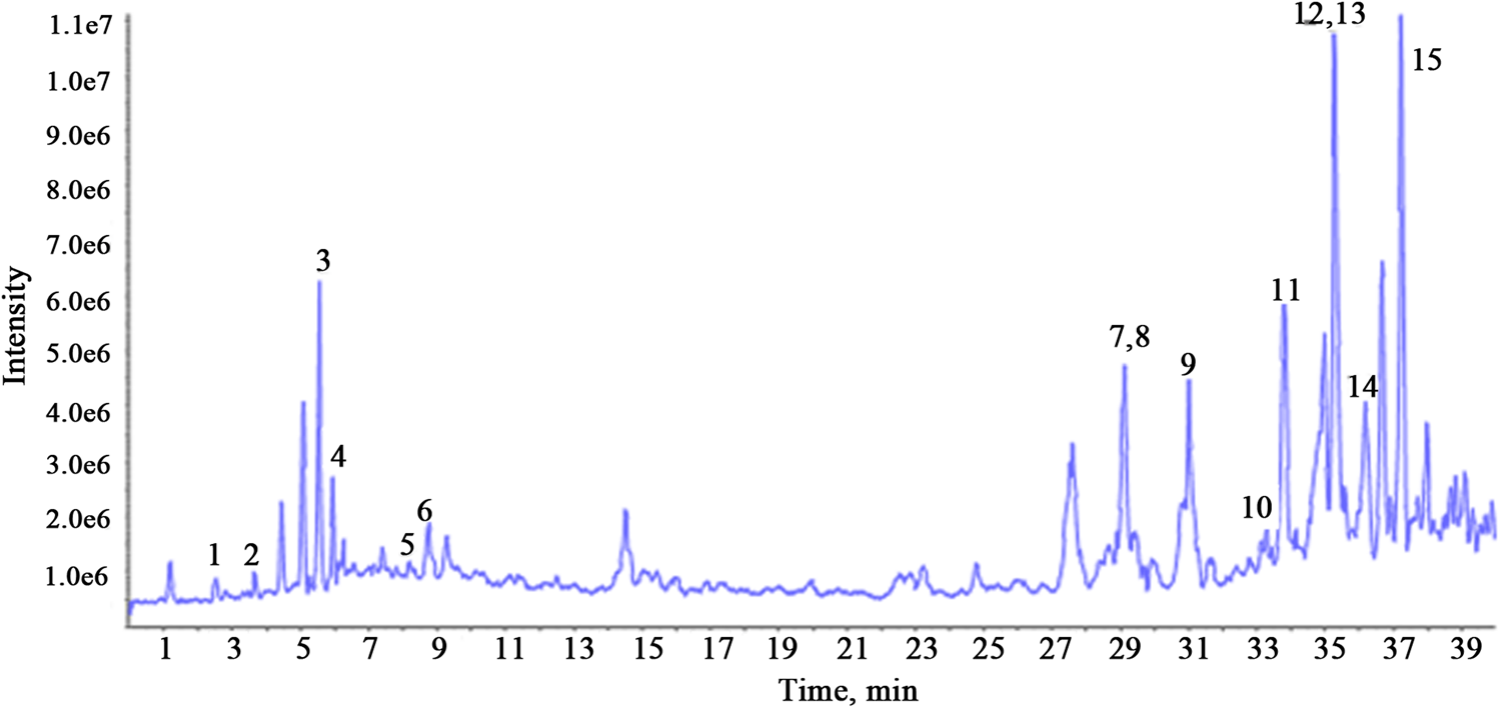
Total ion chromatogram of TWM by UHPLC-Q-TOF-MS/MS in a positive ion mode.

Components of TWM were analyzed by the Triple TOF™ 5600 + system. The total ion chromatogram in a positive ion mode is shown in Figure 1. Fifteen compounds were identified by comparing the MS/MS characteristics and the retention time (Table 1). Wilforgine and wilfordine, except for TP, also have hepatotoxicity in TWM. These components can be used as P-glycoprotein substrates to competitively inhibit the excretion of TP to bile through P-glycoprotein, leading to the accumulation of TP in the liver, which results in the hepatotoxicity [13].

**Table 1:** Main compounds in TWM identified by UHPLC-Q-TOF-MS/MS

Peaks	*t*R (min)	Molecular formula	Product ion	*m*/*z*	Fragment ions	Compounds
1	2.5	C_21_H_27_O_3_N_3_	[M + H]^+^	370.2125	249.1213, 166.0847, 160.1108	Celafurine
2	3.49	C_23_H_29_O_2_N_3_	[M + H]^+^	380.2333	176.1049, 160.1104	Celabazine
3	5.48	C_25_H_31_O_2_N_3_	[M + H]^+^	406.2489	258.1936, 202.1207, 160.1102, 131.0477	Celacinnine
4	5.94	C_30_H_44_O_3_	[M + H]^+^	453.3362	435.3303, 322.2473, 209.1636,114.0907	Wilforlide B
5	8.41	C_20_H_24_O_6_	[M + H]^+^	361.1645	315.2420, 223.1104, 201.0915, 157.0989, 145.0989, 129.0672, 121.0649	Triptolide
6	8.73	C_36_H_45_O_18_N	[M + H]^+^	780.2709	762.2518, 752.2670, 194.0789	Wilforidine
7	29.1	C_41_H_47_O_20_N	[M + H]^+^	874.2764	856.2573, 846.2717, 828.2633, 674.2386	Wilfordconine
8	29.41	C_20_H_28_O_3_	[M + H]^+^	317.2111	281.1883, 9.1425, 215.1412, 189.1267, 147.0790	12,14-Dihydroxy-3-oxo-abieta-8,11,13-triene
9	31.03	C_38_H_47_O_18_N	[M + H]^+^	806.2866	788.2701, 778.2862, 704.2498, 206.0796	Euonymine
10	33.28	C_39_H_45_O_18_N	[M + H]^+^	816.2709	798.2540, 206.0801, 178.0849	1-Desacetylwilforgine
11	33.8	C_43_H_49_NO_19_	[M + H]^+^	884.2972	866.2814, 856.2954, 838.2866	Wtlfordine
12	35.26	C_41_H_47_O_19_N	[M + H]^+^	858.2815	840.2633, 798.2544, 686.2387, 704.2501, 206.0800, 178.0851	Wilforgine
13	35.38	C_38_H_47_O_18_N	[M + H]^+^	806.2866	788.2681, 728.2488, 686.2387, 206.0798	Wilformine
14	36.18	C_41_H_47_O_17_N	[M + H]^+^	826.2917	808.2743, 206.0796, 78.0849	Wilforzine
15	37.23	C_43_H_49_O_18_N	[M + H]^+^	868.3022	850.2846, 746.2603, 686.2387, 704.2495, 206.0802, 178.0851	Wilfordine

### 3.2 Biochemical parameters and histopathological observations

**Figure 2:**
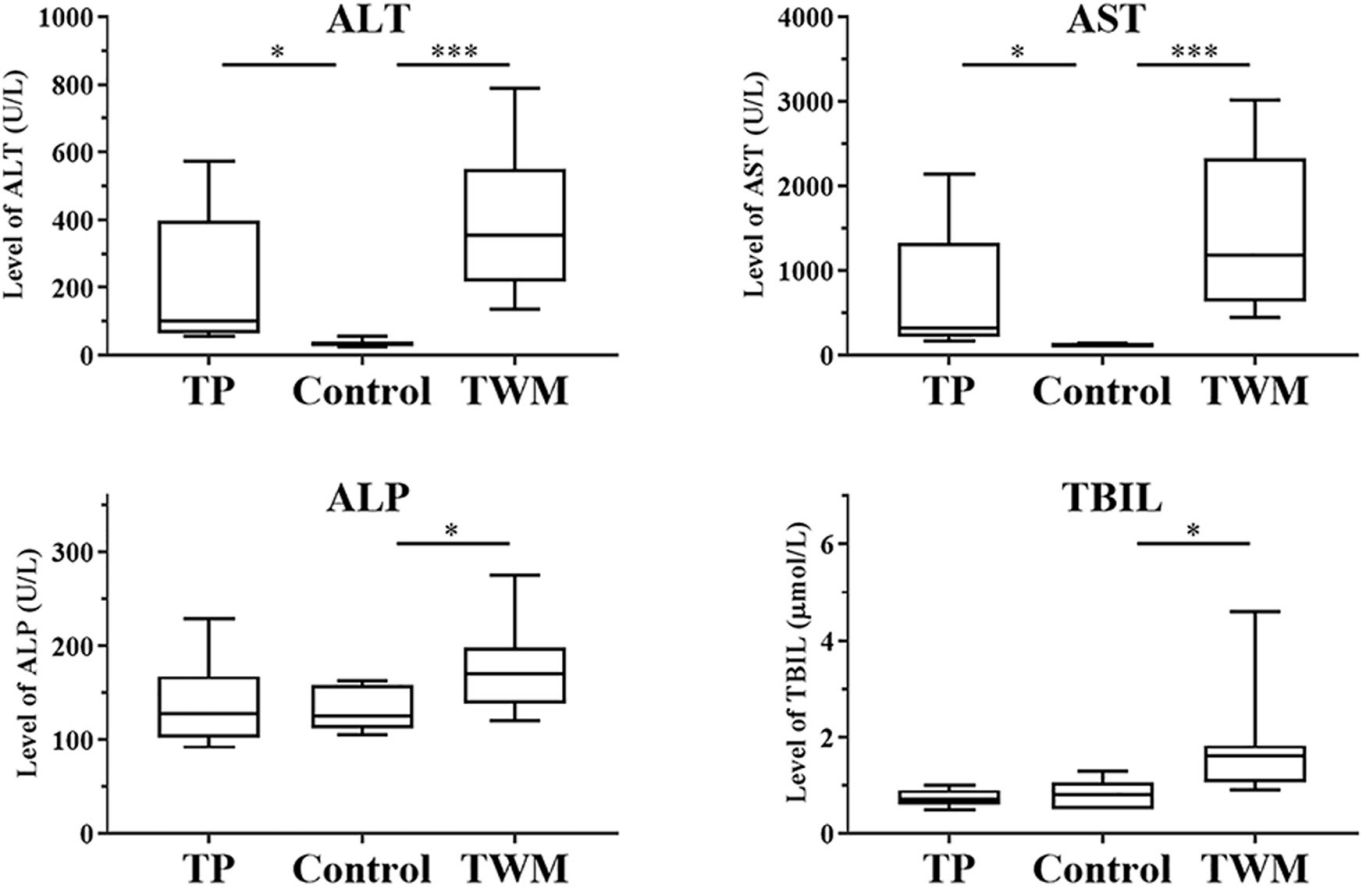
Assay of biochemical indicators in the serum of TWM- and TP-induced liver injured rats. *P < 0.05, **P < 0.01 and ***P < 0.001, significantly different from the control group.

**Figure 3:**
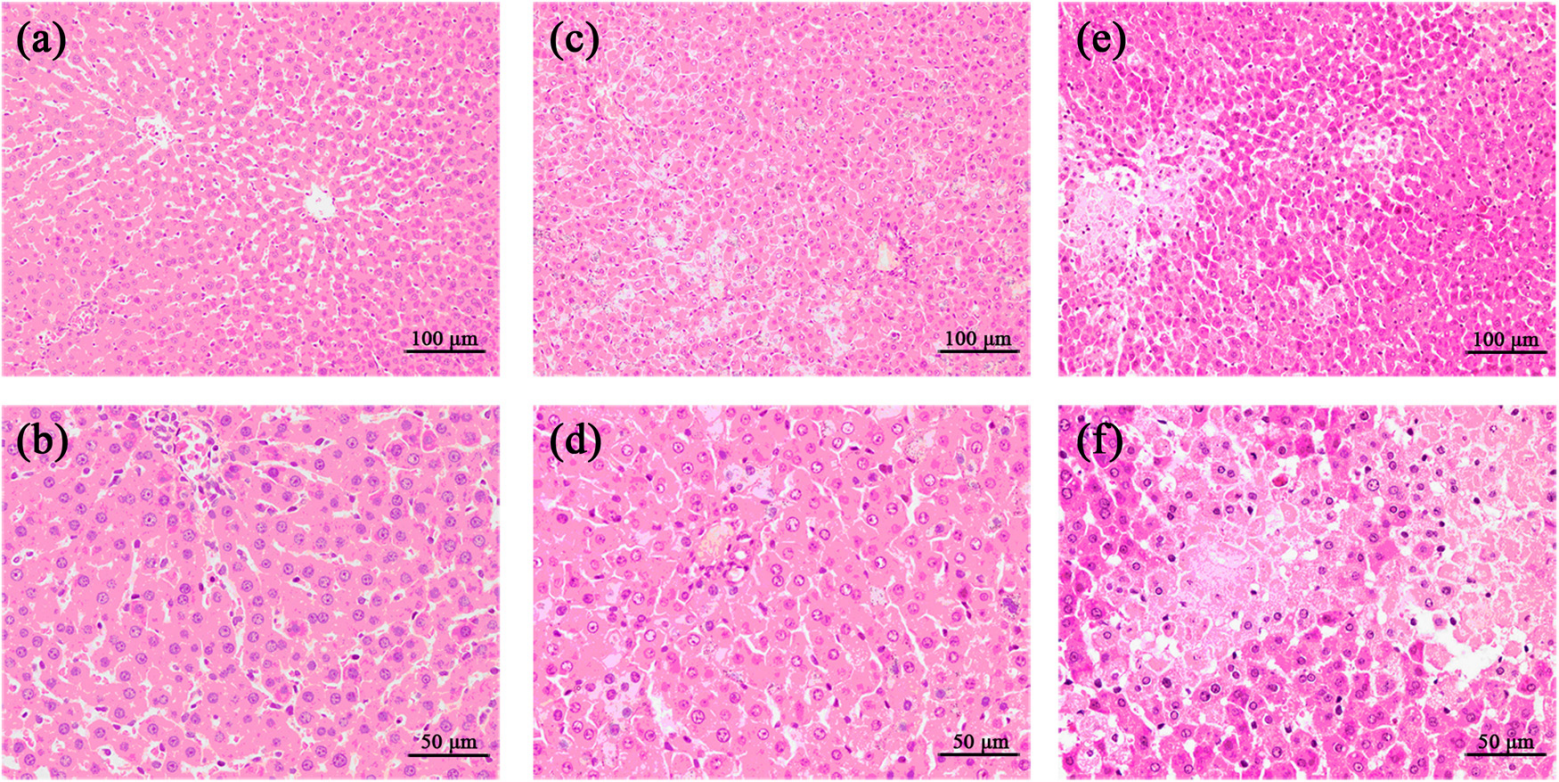
Histopathology of liver tissue 24 h after TWM and TP administration. Representative slides are shown at two magnifications (200× and 400×). (a/b), control group; (c/d), TP-treated group; (e/f), TWM-treated group.

The results of serum biochemical indicators determined by Roche COBAS C501 automatic biochemical analyzer are shown in Figure 2. TWM-treated rats had a significant increase in the serum levels of all biochemical indicators including ALT, AST, ALP and TBIL when compared with control rats (*p* < 0.001 and *p* < 0.05). The serum levels of ALT and AST in TP-treated rats significantly elevated compared to the control rats (*p* < 0.05). The histopathology of livers was also examined 24 h post-TWM and TP administration. As shown in Figure 3, control rats displayed normal liver histology, whereas TWM-treated rats showed obvious rupture of cell membrane, nucleus shrinkage and hyperchromatism, as well as TP-treated rats showed nucleus dissolution, fragmentation and cell vacuolization. The elevated serum levels of biochemical indicators and histopathological alterations all indicated progressive liver injury 24 h after TWM and TP administration.

### 3.3 GC-MS analysis of fatty acids in rat serum

Sixteen fatty acids in rat serum were simultaneously quantified by the GC-MS method. The concentrations of all free and esterified fatty acids were computed using standard curves for the corresponding references. The determined levels of serum fatty acids are summarized in Table 2. These data showed that the lower levels of serum fatty acids were detected in the TWM- and TP-treated rats when compared with control rats. Serum concentrations of all free fatty acids exception for C12:0 and C20:3n6 were significantly decreased in the TP-treated group when compared with that in the control group. Serum concentrations of free fatty acids including C16:0, C18:0, C20:4n6, C22:0 and C22:6n3 were noticeably decreased and that of C20:3n6 was noticeably increased in the TWM-treated group when compared with that in the control group. Importantly, the serum level of C16:0 in the TP-treated groups downregulated ∼3 folds. In addition, the serum levels of esterified fatty acids including C12:0, C14:0, C16:0, C18:0, C18:2n6, C18:3n6, C20:4n6, C20:5n, C22:0 and C22:6n3 in the TP-treated group and C18:0, C20:4n6, C20:5n3, C22:0 and C22:6n3 in the TWM-treated group were significantly decreased when compared with that of the control group. Moreover, the serum level of C20:4n6 in the TWM-treated group downregulated ∼2 folds.

**Table 2:** Serum concentrations of free and esterified fatty acids derived from control, TP-treated and TWM-treated rats measured by GC-MS

Fatty acids		Control	TP treated	TWM treated
Free fatty acids	C12:0	0.42 ± 0.09	0.38 ± 0.12	0.51 ± 0.20
	C14:0	1.67 ± 0.77	0.71 ± 0.37*	1.48 ± 0.91
	C16:0	71.24 ± 18.71	27.96 ± 8.12***	46.85 ± 13.71*
	C16:1n7	2.46 ± 0.51	1.47 ± 0.40***	2.61 ± 1.05
	C18:0	91.51 ± 22.09	49.9 ± 9.13***	53.12 ± 9.37**
	C18:1n9	33.22 ± 5.12	24.7 ± 7.03*	38.33 ± 10.81
	C18:1n7	5.27 ± 0.82	3.32 ± 0.61***	4.95 ± 1.26
	C18:2n6	76.7 ± 13.76	44.93 ± 9.11***	63.10 ± 14.59
	C18:3n6	7.43 ± 0.12	7.18 ± 0.08***	7.38 ± 0.15
	C18:3n3	8.55 ± 0.39	7.87 ± 0.39**	8.24 ± 0.37
	C20:0	1.47 ± 0.06	1.38 ± 0.06**	1.43 ± 0.17
	C20:3n6	7.77 ± 0.31	8.10 ± 0.68	8.75 ± 0.62**
	C20:4n6	76.87 ± 15.06	44.56 ± 8.43***	50.70 ± 9.22***
	C20:5n	17.61 ± 1.08	16.00 ± 0.82**	17.07 ± 0.76
	C22:0	8.11 ± 0.04	8.07 ± 0.05**	8.08 ± 0.22*
	C22:6n3	162.74 ± 24.78	111.24 ± 19.48***	135.96 ± 17.05*
Esterified fatty acids	C12:0	0.12 ± 0.05	0.05 ± 0.03**	0.30 ± 0.21*
	C14:0	1.74 ± 0.50	1.12 ± 0.51*	2.74 ± 1.65
	C16:0	161.63 ± 32.55	88.30 ± 29.37***	125.97 ± 44.7
	C16:1n7	3.58 ± 0.78	2.98 ± 1.13	4.93 ± 1.90
	C18:0	155.81 ± 22.67	102.67 ± 26.91**	119.31 ± 28.59**
	C18:1n9	70.31 ± 20.45	70.9 ± 33.68	89.87 ± 28.23
	C18:1n7	7.84 ± 2.14	5.64 ± 3.33	8.71 ± 4.84
	C18:2n6	198.83 ± 45.29	128.34 ± 43.52**	174.31 ± 77.43
	C18:3n6	3.35 ± 0.62	2.27 ± 0.46**	2.80 ± 1.04
	C18:3n3	4.59 ± 1.12	4.09 ± 1.10	4.81 ± 1.71
	C20:0	3.61 ± 0.10	3.51 ± 0.13	3.54 ± 0.23
	C20:3n6	11.16 ± 0.90	13.45 ± 3.77	13.37 ± 3.04
	C20:4n6	353.62 ± 41.43	194.6 ± 49.08***	179.28 ± 29.58***
	C20:5n3	32.55 ± 7.90	19.27 ± 5.53**	20.95 ± 5.94**
	C22:0	5.83 ± 0.08	5.70 ± 0.07***	5.68 ± 0.06**
	C22:6n3	425.97 ± 55.24	241.62 ± 96.4***	278.46 ± 93.7**

Note: Values are expressed in μg/mL as mean ± standard deviation. *P < 0.05, **P < 0.01 and ***P < 0.001, significantly different from the control group.

### 3.4 GC-MS analysis of fatty acids in rat liver tissue

**Figure 4:**
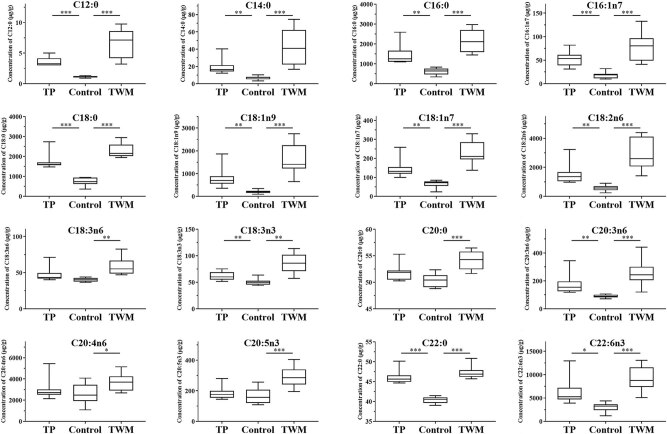
Assay of free fatty acids in the liver of TP-treated and TWM-treated rats. *P < 0.05, **P < 0.01 and ***P < 0.001, significantly different from the control group.

**Figure 5:**
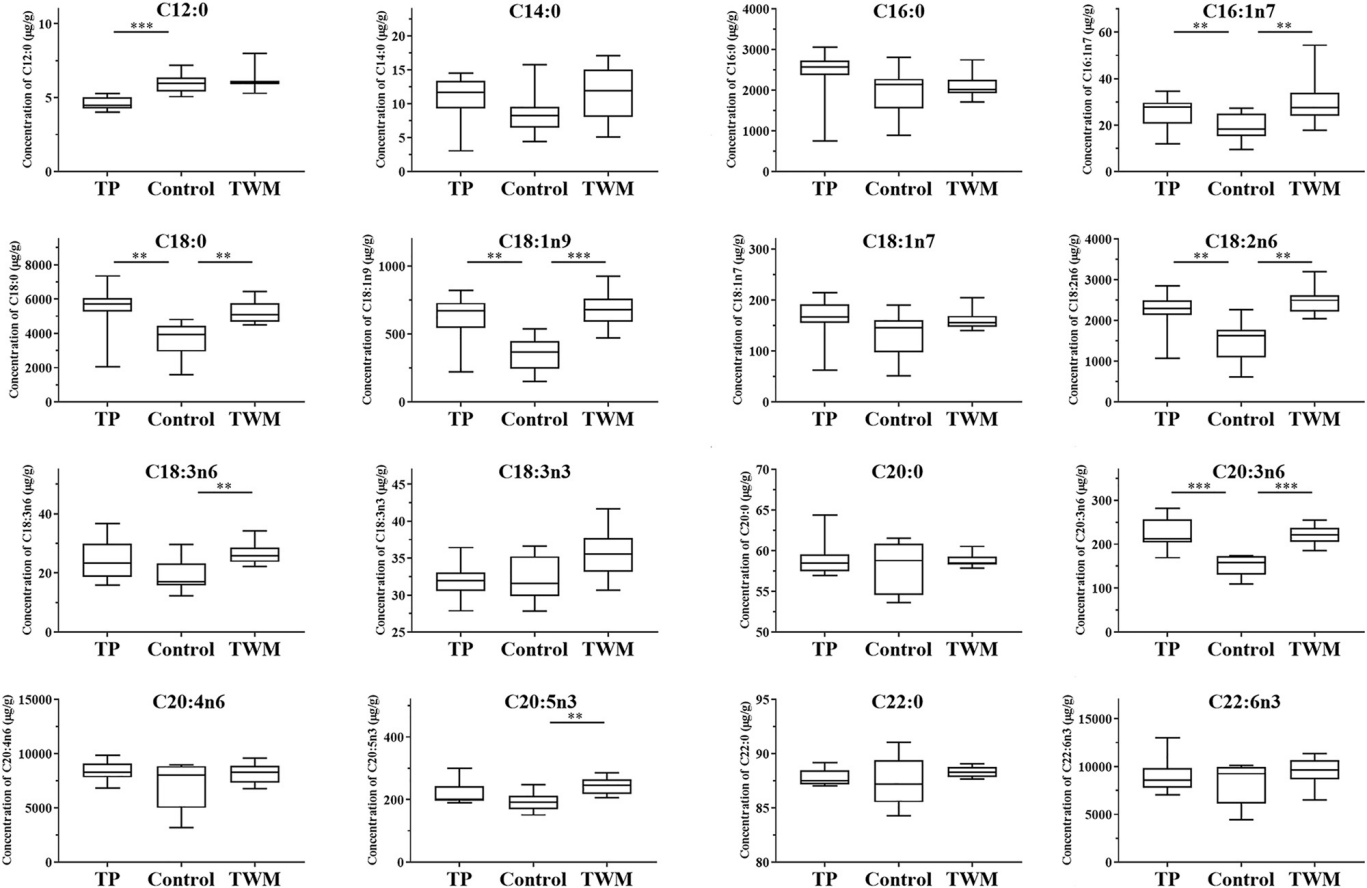
Assay of esterified fatty acids in the liver of TP-treated and TWM-treated rats. *P < 0.05, **P < 0.01 and ***P < 0.001, significantly different from the control group.

The determined levels of free fatty acids and esterified fatty acids in the liver tissue are summarized in Table A1. These data indicated that the higher levels of fatty acids in liver tissue were observed in TWM- and TP-treated groups when compared with the control group. As shown in Figure 4, the concentrations of all determined free fatty acids except C18:3n6, C20:0 and C20:5n3 in TP-treated rats and all determined free fatty acids in TWM-treated rats were significantly higher than those of control rats. More importantly, the levels of C16:0, C18:0, C18:1n9, C18:1n7, C18:2n6 and C22:6n3 in TWM- and TP-treated rats have been upregulated 2–5 folds. As shown in Figure 5, the concentrations of esterified fatty acids including C16:1n7, C18:0, C18:1n9, C18:2n6 and C20:3n in the TP-treated group and C16:1n7, C18:0, C18:1n9, C18:2n6, C18:3n6, C20:3n6 and C20:5n3 in the TWM-treated group significantly increased when compared with the control group.

### 3.5 Multivariate statistical analysis

**Figure 6:**
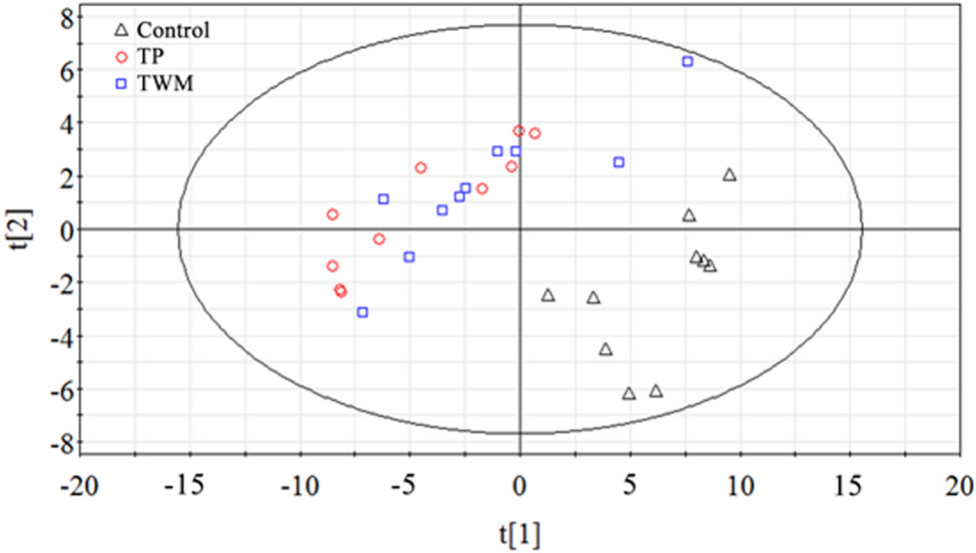
PCA score plot derived from three representative control (Δ), TWM-treated (□) and TP-treated (○) groups using the metabolic profiles of serum free and esterified fatty acids. Two PCs score visualization for sample types. The score plot showed an obvious separation between control and two toxic groups.

**Figure A2:**
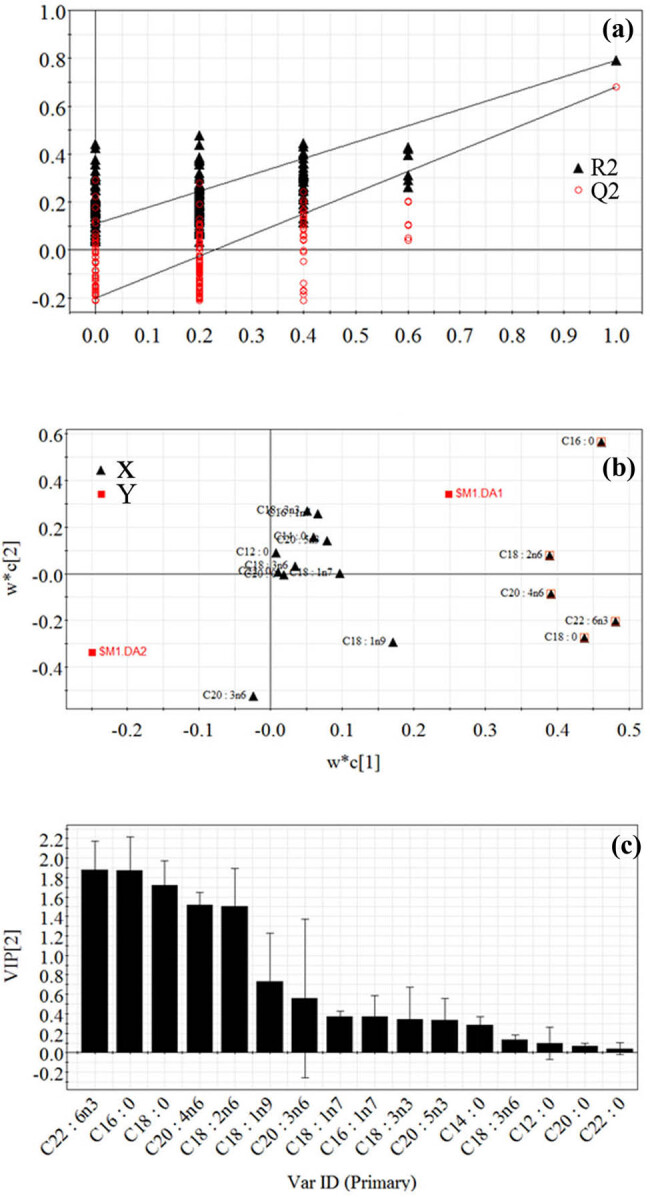
Analysis of candidate biomarkers of TP-induced liver injury using PLS-DA. The PLS-DA model (*R* ^2^ *X* = 92.1%, *R* ^2^ *Y* = 79.2%, *Q* ^2^ = 68.1%) was derived from control and TP-treated groups using the metabolic profile of free fatty acids. (a) The result of the permutation test (*n* = 200) indicated that the original PLS-DA model was valid. (b) The loading plot exhibited that the TP-induced hepatic injury was highly correlated with C16:0, C18:0, C18:2n6, C20:4n6 and C22:6n3 marked with “□.” (c) VIP plot verified the importance of these fatty acids with VIP > 1.0 in classification.

**Figure A3:**
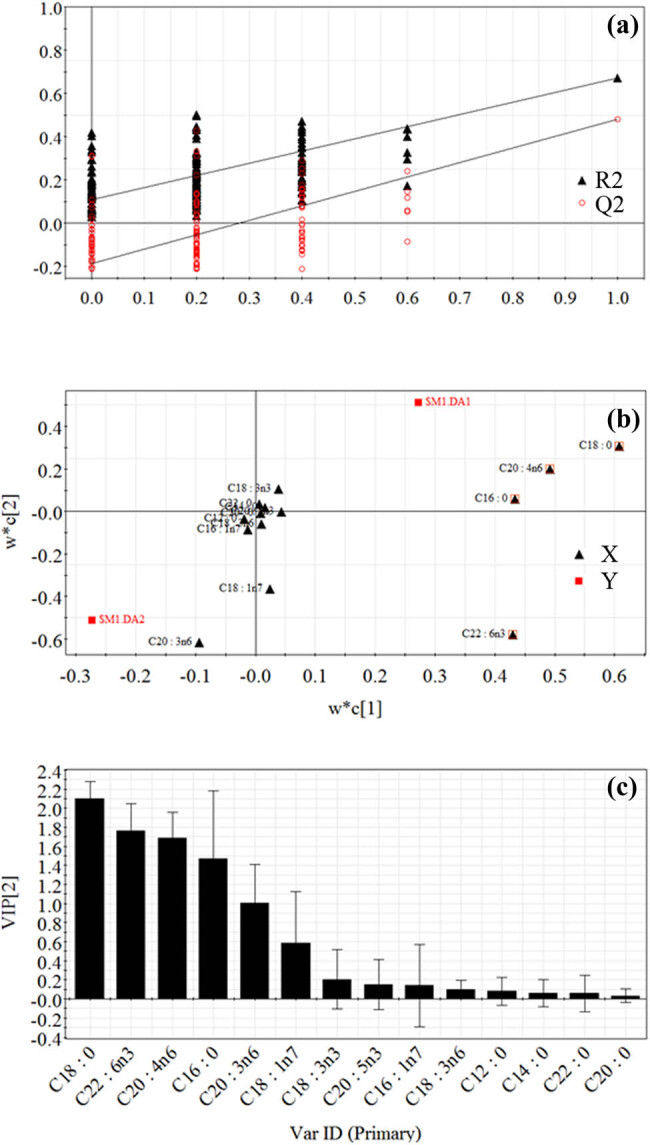
Permutation test plot (*n* = 200) (a), loading plot (b) and VIP plot (c) in the PLS-DA model (*R* ^2^ *X* = 87.5%, *R* ^2^ *Y* = 67.1% and *Q* ^2^ = 48%) derived from control and TWM-treated groups using the serum free fatty acid metabolic profile. C16:0, C18:0, C20:4n6 and C22:6n3 were identified as potential biomarkers of TWM-induced liver injury.

PCA and PLS-DA were used to investigate the metabolic profiles of serum fatty acids for TMW- and TP-induced liver injury. Thirty-two variables including 16 free and 16 esterified fatty acids in serum were first analyzed by PCA of two principal components (PC1 and PC2). The two PCs explained 83.1% of the systematic variation. As shown in the two-dimension score plot (Figure 6), TWM- and TP-treated groups were combined together, and both of them could be clearly distinguished from the control group. The PCA result showed that the serum fatty acid metabolic pattern was different between control and two toxic groups. A previous study by our group indicated that serum free fatty acids could be promising biomarkers of DILI since these species play key roles in many metabolic processes [3]. Thus, 16 determined serum free fatty acids as X variables were employed to construct PLS-DA models to identify potential biomarkers of TWM- and TP-induced liver injury. The first PLS-DA model was constructed by serum samples from the control and the TP-treated group. Values of *R* ^2^ *X*, *R* ^2^ *Y* and *Q* ^2^ in the model were 92.1, 79.2 and 68.1%, respectively, which indicated that the model has good explanatory ability to variables (including *X* and *Y*) and good predictive ability to the model. The model was further verified by permutation test (*n* = 200). As shown in Figure A2-a, all red *Q* ^2^-values were lower than the original points to the right, and the black regression line of the *Q* ^2^-points intersected the vertical axis below zero, which demonstrated that the original PLS-DA model was valid. Loading plot (Figure A2-b) and variable importance in the projection (VIP) values (Figure A2-c) showed that the most important variables on classification were considered as C16:0, C18:0, C18:2n6, C20:4n6 and C22:6n3. The second PLS-DA model from control and TWM-treated groups was similarly constructed. However, there was no significant difference in the serum levels of C18:1*n*9 and C18:2*n*6 with VIP > 1.0 in the PLS-DA model between control and TWM-treated groups. Therefore, the second PLS-DA model (*R* ^2^ *X* = 87.5%, *R* ^2^ *Y* = 67.1% and *Q* ^2^ = 48%) was reconstructed after the two species were eliminated. This measure could avoid erroneous conclusion by PLS-DA. Figure A3 shows that the most important variables on classification were considered as C16:0, C18:0, C20:4*n*6 and C22:6*n*3. In short, the above analysis suggested that four free fatty acids (including C16:0, C18:0, C20:4*n*6 and C22:6*n*3) were identified as common potential biomarkers of TP- and TWM-induced liver injury. Pearson’s correlation analysis was applied to measure the correlations between serum concentrations of the four candidate fatty acid biomarkers (including free and esterified fatty acids) and the activities of ALT and AST that currently served as clinical indicators of hepatic damage. Pearson’s correlation analysis result showed that all correlation coefficients (*r*) were lower than zero, suggesting that these four fatty acids were negatively correlated with the two aminotransferases (Table A2). Moreover, free and esterified fatty acids for C20:4n6 in TWM-induced liver injury were strongly correlated with the two aminotransferases (*r* < −0.7). Altogether, chemometric and correlation analyses demonstrated that the four fatty acid biomarkers and their metabolic profiling could be used as promising diagnostic indicators for TWM- and TP-caused hepatic damage.

## 4 Discussion

Except for reproductive toxicity, other toxicities such as hepatotoxicity and nephrotoxicity showed gender differences. Due to specifically expressed CYP3A2 in male rats resulting in acceleration of TP metabolism, TP-treated female rats exhibited greater toxicity when compared to male rats [20]. Therefore, female rats were used to develop TP- and TWM-induced liver-injured rat models based on the study’s aim on the correlation between fatty acid metabolic profiling and TP-/TWM-caused liver injury.

Fatty acid levels in serum and liver tissues are sensitively influenced by chemical liver injury, and thus, we study this level changes to show the damage in the liver. In this article, the presented concentration data showed an opposite change trend of serum and liver fatty acid levels after TWM or TP administration: a decrease in serum levels and an increase in levels of fatty acids in the liver including free and esterified fatty acids. β-Oxidation, the most important metabolic pathway for fatty acids, is regulated by l-carnitine and rate-limiting enzyme carnitine palmitoyltransferase I (CPT I), which was responsible for fatty acyl CoA transport from cytoplasm to mitochondria. Moreover, peroxisome proliferator-activated nuclear receptor (PPARα) can adjust the activity of CPT I [21,22]. β-Oxidation of fatty acids in testicle was irreversibly restrained after TP administration by decreasing the levels of l-carnitine, acetyl-l-carnitine and PPARα protein expression, resulting in the accumulation of free fatty acids in the testicular tissue [23]. This study offered further proof that TWM and TP could inhibit the β-oxidation of fatty acids in the liver according to the significantly increased levels of free and esterified fatty acids in the liver of TWM- and TP-treated rats. Oxidative stress injury caused by reactive oxygen species was one of the important mechanisms of TWM and TP-induced liver toxicity. Lipid peroxidation was induced by oxidative stress injury in TWM- and TP-treated rats, leading to a significant decrease in serum levels of most of fatty acids, especially polyunsaturated fatty acids.

C20:4n6, a ω-6 polyunsaturated fatty acid, is a precursor of many active endogenous substances, such as various inflammatory factors, in the body. C20:4n6-related inflammatory metabolic pathway was regulated by phospholipase A2 (PLA2), the activity of which was positively correlated with Ca^2+^ concentration in the hepatic cell. The significant elevated level of Ca^2+^ in hepatocyte was determined in TP-induced liver-injured rats [24,25]. Therefore, we speculated that overdose TP could induce C20:4n6-related inflammatory metabolic pathway, resulting in the significant decreased level of C20:4n6 in the liver. However, in this study, the level of C20:4n6 in liver was not significantly different between the TP-treated rats and controls, which may be the combined result of fatty acid β-oxidation inhibition and PLA2 activity upregulation. C22:6n3, a ω-3 polyunsaturated fatty acid, could inhibit the activities of key enzymes in C20:4n6-related inflammatory metabolic pathway such as PLA2 and cyclooxygenase 2, which indicated that C22:6n3 had a strong anti-inflammatory effect [26]. Moreover, C22:6n3 could increase the expression level of CPT І by upregulating the PPARα activity to accelerate the β-oxidation of fatty acids [27]. C16:0 and C18:0 are the most important long-chain saturated fatty acids for their higher concentrations *in vivo*. Nutrition research indicated that the serum level of saturated free fatty acids was closely correlated with the development of nonalcoholic fatty liver disease [28]. C16:0 could induce oxidation stress in the hepatic cell *in vitro* via increased CD36 expression [29], and it was positively correlated with inflammation. Contrarily, C18:0 was considered a potent anti-inflammatory lipid [30]. Altogether, the four serum free fatty acids including C16:0, C18:0, C20:4n6 and C22:6n3 played a crucial role in the development of liver injury caused by TWM and TP, and were identified as potential biomarkers of TWM- and TP-induced liver injury.

## 5 Conclusion

In the study, TWM- and TP-treated rats can be obviously differentiated from control rats by PCA and PLS-DA based on the metabolic profiling of fatty acids in serum. Serum free fatty acids for C16:0, C18:0, C20:4n6 and C22:6n3 were considered as potential biomarkers of TWM- and TP-induced liver injury. Moreover, C20:4n6 in the serum of TWM-induced liver injured rats exhibited a high negative correlation (*r* < −0.7) with ALT and AST levels. Altogether, TWM- and TP-induced liver injuries were closely related to the metabolic profiling of serum fatty acids. It clearly stated that a novel metabolomic method based on the serum fatty acid metabolic profiling could be a supplementary approach on DILI diagnosis in clinic.

## Appendix

**Table A1:** Concentrations of free and esterified fatty acids in liver tissue derived from control, TP-treated and TWM-treated rats measured by GC-MS

Fatty acids		Control	TP treated	TWM treated
Free fatty acids	C12:0	1.13 ± 0.14	3.62 ± 0.74***	6.69 ± 2.22***
	C14:0	6.36 ± 2.03	19.53 ± 8.89**	42.74 ± 20.39***
	C16:0	616.39 ± 159.17	1445.89 ± 468.67**	2162.29 ± 569.93***
	C16:1n7	18.19 ± 6.78	52.06 ± 15.44***	78.68 ± 28.23***
	C18:0	744.20 ± 194.20	1723.37 ± 369.15***	2299.57 ± 362.19***
	C18:1n9	201.80 ± 71.02	801.93 ± 437.16**	1605.99 ± 634.54***
	C18:1n7	65.30 ± 18.42	144.47 ± 44.4**	228.68 ± 59.43***
	C18:2n6	557.57 ± 192.52	1514.95 ± 673.77**	2870.92 ± 1055.04***
	C18:3n6	40.01 ± 2.55	47.35 ± 9.38	59.02 ± 13.06**
	C18:3n3	50.37 ± 5.77	61.56 ± 8.02**	86.78 ± 18.20***
	C20:0	50.45 ± 1.23	51.81 ± 1.44	54.12 ± 1.79***
	C20:3n6	90.01 ± 11.29	175.79 ± 67.37**	257.38 ± 88.27***
	C20:4n6	2642.14 ± 944.11	2956.26 ± 921.54	3662.93 ± 817.06*
	C20:5n3	167.52 ± 48.90	184.35 ± 38.90	290.60 ± 66.67***
	C22:0	40.37 ± 0.81	46.12 ± 1.60***	47.29 ± 1.47***
	C22:6n3	3041.03 ± 913.41	6288.49 ± 2679.29*	9199.99 ± 2654.3***
Esterified fatty acids	C12:0	5.95 ± 0.66	4.57 ± 0.41***	6.13 ± 0.71
	C14:0	8.51 ± 3.16	10.90 ± 3.45	11.46 ± 4.05
	C16:0	1959.14 ± 574.3	2415.11 ± 629.3	2087.04 ± 283.09
	C16:1n7	19.00 ± 5.96	25.42 ± 6.80**	29.81 ± 10.07**
	C18:0	3625.7 ± 1037.44	5488.39 ± 1357.17**	5204.89 ± 624.77**
	C18:1n9	348.06 ± 122.00	618.73 ± 188.34**	680.23 ± 123.57***
	C18:1n7	130.27 ± 43.63	164.23 ± 41.95	160.76 ± 19.57
	C18:2n6	1480.48 ± 488.92	2247.08 ± 482.43**	2488.29 ± 324.63**
	C18:3n6	19.00 ± 5.11	24.06 ± 6.68	26.51 ± 3.55**
	C18:3n3	32.31 ± 3.18	31.86 ± 2.39	35.59 ± 3.35
	C20:0	58.11 ± 2.96	58.99 ± 2.16	58.78 ± 0.77
	C20:3n6	152.8 ± 22.27	224.97 ± 35.75***	220.11 ± 21.86***
	C20:4n6	7178.68 ± 2078.04	8356.28 ± 877.79	8197.38 ± 920.08
	C20:5n3	193.77 ± 32.85	220.21 ± 35.58	242.90 ± 27.25**
	C22:0	87.46 ± 2.32	87.83 ± 0.81	88.31 ± 0.49
	C22:6n3	8341.72 ± 2057.19	8936.35 ± 1744.18	9403.66 ± 1406.34

Note: values are expressed in μg/g as mean ± standard deviation. **P* < 0.05, ***P* < 0.01 and ****P* < 0.001, significantly different from the control group.

**Table A2:** Pearson’s correlation analysis between the serum levels of fatty acid biomarkers and two aminotransferases derived from control, TP-treated and TWM-treated rats

		TP (ALT)		TP (AST)		TWM (AST)		TWM (AST)	
Fatty acid		*r*	*p*	*R*	*p*	*r*	*p*	*r*	*p*
Free fatty acids	C22:6n3	−0.479	0.038	−0.473	0.041	−0.592	0.006	−0.586	0.007
	C20:4n6	−0.481	0.037	−0.470	0.042	−0.734	0.000	−0.708	0.000
	C16:0	−0.500	0.029	−0.529	0.020	−0.388	0.091	−0.438	0.054
	C18:0	−0.420	0.074	−0.410	0.081	−0.661	0.002	−0.659	0.002
Esterified fatty acids	C22:6n3	−0.404	0.086	−0.396	0.093	−0.670	0.001	−0.654	0.002
	C20:4n6	−0.408	0.083	−0.400	0.090	−0.792	0.000	−0.791	0.000
	C16:0	−0.514	0.024	−0.507	0.027	−0.486	0.030	−0.456	0.043
	C18:0	−0.449	0.054	−0.436	0.062	−0.676	0.001	−0.681	0.001

*r*, correlation coefficient. The value of *r* close to 1 indicates perfect correlation. Correlation significance was defined as **P* < 0.05, ***P* < 0.01 and ****P* < 0.001.
